# Melanin production and laccase mediated oxidative stress alleviation during fungal-fungal interaction among basidiomycete fungi

**DOI:** 10.1186/s43008-021-00082-y

**Published:** 2021-11-09

**Authors:** Samim Dullah, Dibya Jyoti Hazarika, Gunajit Goswami, Tanushree Borgohain, Alokesh Ghosh, Madhumita Barooah, Ashok Bhattacharyya, Robin Chandra Boro

**Affiliations:** 1 Department of Agricultural Biotechnology, Assam Agricultural University, Jorhat, Assam 785013 India; 2 DBT-North East Centre for Agricultural Biotechnology, Assam Agricultural University, Jorhat, Assam 785013 India; 3 Department of Plant Pathology, Assam Agricultural University, Jorhat, Assam 785013 India

**Keywords:** Hyphal interactions, Laccase, Melanin, NMR spectroscopy, Superoxide dismutase, ROS, qRT-PCR

## Abstract

Fungal-fungal interaction often leads to the change in metabolite profile of both the interacting fungus which may have potential implication in industry or agriculture. In the present study, we performed two sets of fungal-fungal interaction—*Trametes coccinea* (F3) with *Leiotrametes lactinea* (F9) and *T. coccinea* (F3) with *T. versicolor* (F1) to understand the changes in the metabolite profile during the interaction process and how this process impacts the hyphal/mycelial morphology of the participating fungi. The metabolites produced during interaction of *T. coccinea* (F3) with *L. lactinea* (F9) and *T. coccinea* (F3) with *T. versicolor* (F1) was analysed through liquid chromatography coupled to mass spectroscopy (LC-MS). Most of the metabolites secreted or produced during interaction are associated with defensive response. Further, visualization with scanning electron microscopy revealed that interaction between the tested fungi led to the changes in the hyphal morphology. The bipartite fungal interaction resulted in the production of a dark brown colour pigment—melanin as confirmed by the LC-MS, FTIR and NMR analysis. Moreover, the fungal–fungal interaction also led to increase in the production of laccase, a group of multicopper oxidases involved in detoxification of toxic compounds. Further, increased activity of superoxide dismutase, an enzyme that catalyzes the dismutation of the superoxide anion to hydrogen peroxide was also recorded during fungal–fungal interaction. Quantitative real-time PCR revealed upregulation of *lcc1* (encoding a laccase enzyme) and few other stress related genes of *T. versicolor* during its hyphal interaction with *T. coccinea*, suggesting a direct correlation between laccase production and melanin production.

**Supplementary Information:**

The online version contains supplementary material available at 10.1186/s43008-021-00082-y.

## Introduction

Fungal-fungal interactions are highly dynamic phenomenon which occurs in nature, whereby the interacting fungi compete for available nutritional source and territory. These interactions lead to the induction of an array of bioactive products by stimulating the complex metabolic pathways (Bertrand et al. 2014). Co-culturing fungal species results in the production of some novel metabolites as a response to antagonistic interaction (Chatterjee et al. 2016). Few studies have been carried out to unravel the morphological and enzymatic changes during fungal interaction. During interaction of two different fungal species, various metabolic pathways get induced in the zones of barrage formation. The induction of certain metabolic pathways are due to the production of toxins, growth inhibitors and their by-products (Rodriguez et al. 2011). Phelligridin C, phelligridin H, methyl inoscavin A, inoscavin C, methyl davallialactone and foscoparianol D are some compounds observed during the co-culture of *Inonotus obliquus* and *Phellinus punctatus* (Zheng et al. 2011). During interaction, alcohols, aldehydes, ketones, terpenes, aromatic compounds and reactive oxygen species (ROS) are produced as a result of antagonism (Evans et al. 2008), which leads to the up-regulation of many oxidative enzymes like laccase, manganese peroxidase, lignin peroxidase (Gregorio et al. 2006). For instance, dual culture of *Trichoderma* and *Metarhizium* leads to oxidative stress and production of sugar alcohols in the zone of interaction (Medina et al. 2020). Tamayo et al. (2016) reported that the increase in ROS activated enzymes like superoxide dismutases (SODs) which acted as first line of defense.

Interspecific interaction among wood rotting basidiomycetes is a natural phenomenon of the ecosystem dynamics. For example, *Ganoderma applanatum, Gloeophyllum trabeum*, *Irpex lacteus*, *Pleurotus ostreatus, Trametes coccinea*, and *T. versicolor*, are some of the common wood inhabiting basidiomycetes that occupy the same microhabitat (Song et al. 2012). The outcome of such interactions can be either replacement—where one fungus gains the territory of the other, or deadlock—where neither of the two interacting fungi gains the territory of one another. Interactions among these basidiomycete fungi in the environment may help in decomposing and cycling of nutrients like carbon and nitrogen, thus exhibiting beneficial effects to the environment. Besides environmental utility, the interactions also have numerous industrial application such as induction of industrially important enzymes like laccase, xylanase, cellulase; production of important novel bioactive compounds. Therefore, understanding the basic metabolism and molecular mechanism is essential for their industrial and biotechnological applications. In a previous study, we assigned different grades of interaction during in vitro dual culture of different basidiomycete and ascomycete fungi. Our study revealed that *T. coccinea* (F3), when interacted with *T. versicolor* (F1) and *L. lactinea* (F9), formed a deadlock type of interaction with significant increase in the production of hydrolytic enzymes (Dullah et al. 2021). In the present study, the metabolic responses of the confronting mycelia of these fungal species were assessed during in vitro interspecific interaction of *T. coccinea* with *T. versicolor* and *T. coccinea* with *L. lactinea* using liquid chromatography-mass spectrometry (LC-MS). The interactions of *T*. *coccinea* with *T. versicolor* and *T. coccinea* with *L. lactinea* were chosen based on their morphological studies as well as their promising laccase and superoxide dismutase activity observed during interaction. The study also focuses on analyzing an important natural product-melanin produced during the interaction.

## Materials and methods

### Strains and culture condition

Three previously isolated fungal isolates viz*.*, *Trametes versicolor* F1 (MK370665), *T. coccinea* F3 (MK168589) and *Leiotrametes lactinea* F9 (MK168586) were obtained from the Microbial Biotechnology laboratory, Department of Agricultural Biotechnology, Assam Agricultural University, Jorhat for the interaction studies. These species were characterized previously based on their morphological characters and molecular information of the internal transcribed spacer (ITS) region (Dullah et al. 2021). The cultures were grown on potato dextrose agar (PDA) (Himedia, India) and incubated at 28 °C.

### Interaction study between the fungal isolates

For mono-cultures, a 7 mm agar plug of the fungal culture was inoculated in petri dish containing potato dextrose agar (PDA). *T. coccinea* versus *L. lactinea* and *T. coccinea* versus *T. versicolor* were dual cultured by inoculating 7 mm agar plugs of each isolate onto the opposite sides of a PDA plate and incubated at 28 °C for 15 d.

### Visualization of interacting fungi under scanning electron microscope (SEM)

The hyphae from both the interacting cultures i.e., *T. coccinea* versus *T. versicolor* and *T. coccinea* versus *L. lactinea* and the monocultures on the 9th, 12th and 15th day of incubation were taken and fixed in 2.5% glutaraldehyde (prepared in 0.1 M phosphate buffer), following the protocol of Kathuria et al. (2014). Scanning electron microscopic analysis was done using FEI Quanta 250 SEM at an accelerating voltage of 10 kV with a scanning electron detector for taking micrographs at different magnifications.

### Metabolite extraction for LC-MS

**Fig. 1:**
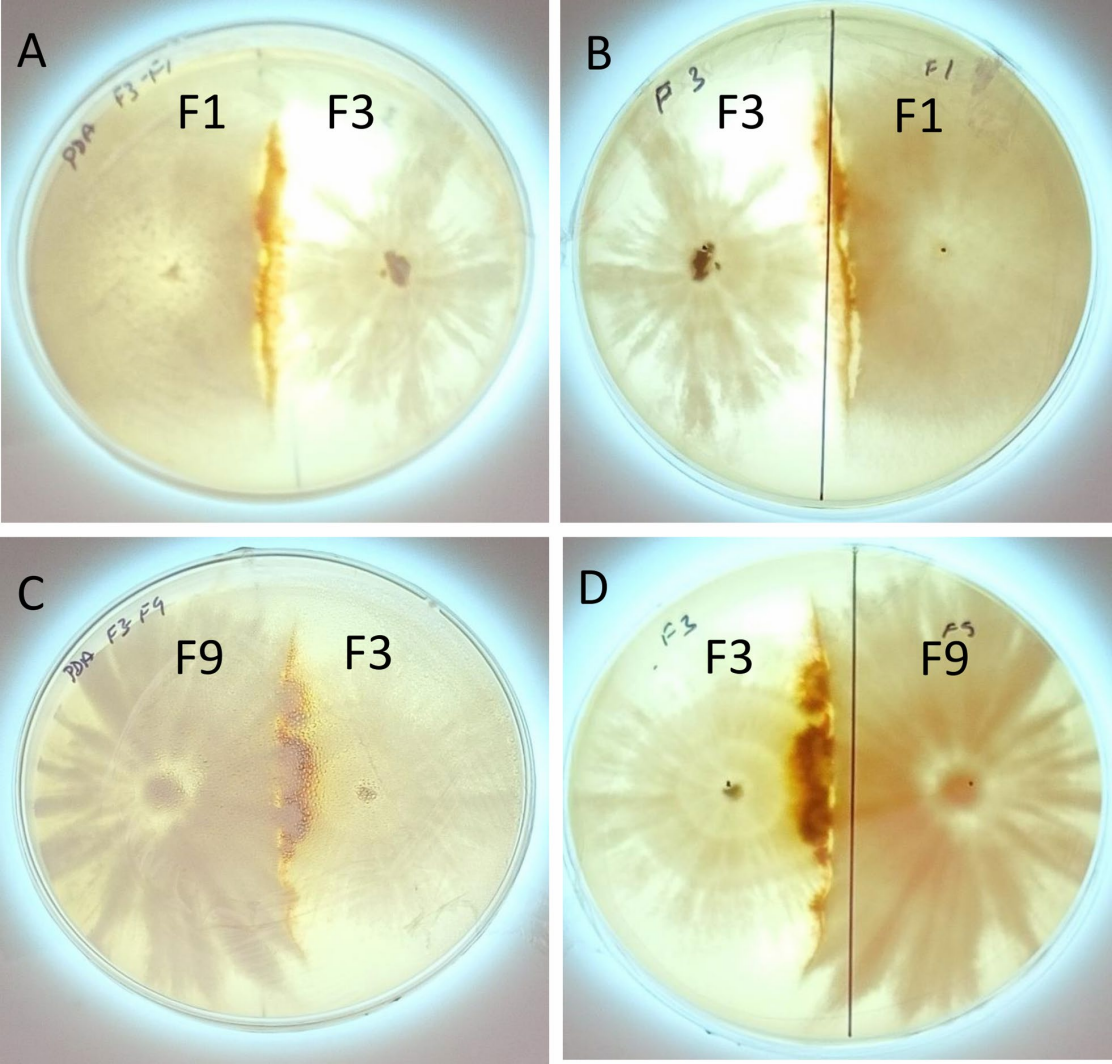
Interspecific interactions of *T. coccinea* with *T. versicolor* and *T. coccinea* with *L. lactinea.* **A**, **B** Front and back view of the interaction between *T. coccinea* and *T. versicolor.* **C**, **D.** Front and back view of the interaction between *T. coccinea* and *L. lactinea*

The two fungal dual cultures (*T. coccinea* with *L. lactinea* and *T. coccinea* with *T. versicolor*) at their 8th day of interaction were considered for metabolite extraction for LC-MS and HRMS (High-Resolution Mass Spectrometry) (Fig. 1). The single cultures of *T. coccinea*, *T. versicolor* and *L. lactinea* were taken as control. The excised mycelia were mixed in a solvent containing methanol: dichloromethane: ethyl acetate in the ratio of 1: 2: 3. It was placed in a rotary shaker at 120 rpm for 12 h and sonicated. The filtrate was dried in a rotary evaporator system (IKA®, Staufen, Germany). The powdered extract of each interaction and monoculture obtained was re-dissolved in methanol. The extract was filtered through 0.22 µm syringe filter. LC-MS was carried out using an Agilent 1260 Infinity HPLC (High-Performance Liquid Chromatography) system (Agilent Technologies, Santa Clara, CA, USA) equipped with an auto sampler. An injection volume of 20 µl was loaded into the system and the separation of individual compounds was carried out through a C18 column [250 length × 4.5 mm internal diameter (ID), pore size: 5 µm] with a gradient elution condition consisting of mobile phase: acetonitrile (solvent A) and 0.01% formic acid in water (solvent B). The gradient profile was as follows: 95% solvent B at 0–5 min, 70% solvent B at 6–11 min, 40% solvent B at 12–19 min, 20% solvent B at 20–25 min, 95% solvent B at 26 min and continued till 30 min. The flow rate was 1.5 ml/min. The separated compounds were ionized using the electrospray ionization (ESI) in the positive mode and detected using the MS detector coupled to the HPLC system. The scan range was from 80 to 1000 m/z. The HRMS analysis was performed in a Xevo G2-XS QT of HRMS system (Waters Corp, Milford, MA, USA) by injecting 10 μl of the extracts, and the positively ionized adduct ions were detected. The molecular weights were confirmed by comparing with the exact mass of compounds obtained from PubChem (NCBI, Maryland, USA).

### Data extraction and compound identification

The peaks from ESI positive chromatogram containing the m/z ratio in one axis and the relative abundance (%) in the other axis for different retention time were analyzed for the presence of different compounds based on the adduct ions formed. The molecular mass for each retention time was predicted by identifying the different adducts. The molecular mass thus obtained were compared with the molecular weight of the reference compounds from different sources including Yeast Metabolome Database, PubChem, NIST molecular database. Some references were specific for a particular species whereas some were based on common metabolites secreted by fungal species.

### Production and extraction of brown pigment

For brown pigment production during interaction, 7 mm mycelial plug from *T. coccinea* versus *L. lactinea* and *T. coccinea* versus *T. versicolor* were inoculated in 100 mL of Sabouraud broth (SB) (Himedia, India). Monocultures of *T. coccinea*, *L. lactinea* and *T. versicolor* were also inoculated and incubated at 28 °C for 21 d. A flask containing media without inoculum was taken as control. When the colour of the broth changed, the mycelial biomass was removed and 1 M NaOH was added to the filtrate making the solution alkaline with pH 10 and autoclaved at 120 °C. The solution was centrifuged at 8000 rpm for 20 min. The filtrate was acidified to pH 2 by adding HCl and centrifuged at 8000 rpm for 15 min. The precipitate was washed with organic solvents and HPLC grade water to remove any acid traces and dried at room temperature. The dried pigment was collected and weighed (Arun et al. 2015; Sun et al. 2017).

### Characterization of brown pigment

The dried pigment was characterized for its solubility in water, 1 M KOH, 1 M NaOH and organic solvents like ethanol, hexane, and acetone. Reactivity of the pigment with hydrogen peroxide was tested by reacting 50 mg melanin with 30% H_2_O_2_, followed by precipitation with 1 mol/L HCl.

### HPLC analysis

The HPLC analysis was done by following the protocol of Sun et al. (2016). The extracted melanin pigment (after purification using TLC) and the commercially available melanin (Sigma, USA) were dissolved in a 0.5 M NaOH solution and filtered with the help of a 0.2 µm filter. The HPLC analysis was performed with a Hitachi Chromaster 3000 series HPLC system (Hitachi, Tokyo, Japan), using a diode array detector (DAD) at 260 nm and a Cosmosil C18 (300 mm length × 4.6 mm ID, pore size: 5 µm) column. The mobile phase consisted of methanol and acetic acid (ratio of 99:1) in an isocratic elution for 30 min (flow rate: 0.5 ml/min, injection volume: 10 µl, and column temperature: 25 °C). The purity of melanin was calculated from the area under the curve of the HPLC chromatogram. The experiment was repeated thrice with independent preparations.

### FTIR and NMR analysis

For fourier-transform infrared spectroscopy (FTIR) analysis, two milligrams of the dried pigment were ground properly with infra-red grade KBr (1:10) and pressed into a disc under vacuum (Al Khatib et al. 2018). The spectrum from 4500 to 400 cm^−1^ was recorded in a Perkin Elmer Inc. spectrophotometer. The pigment composition and structure were predicted by searching the spectrum against a database of reference spectra.

For proton nuclear magnetic resonance spectroscopy (NMR) analysis, the method of Kurian and Bhat (2017) was followed. The pigment was dissolved in deuterated DMSO and the solution was subjected to ^1^H NMR analysis using a Bruker 300 MHz instrument with a magnetic field of 11.4 Tesla.

### Laccase assay

Qualitative screening was done by inoculating a 5 mm diameter mycelial disc of 5-day old culture onto PDA plates containing 4 mM guaiacol (Bodke et al. 2012). Intense brown coloration around the fungal colony was considered positive for laccase production.

For laccase quantification, a mycelial disc from each of the interacting fungi was inoculated into a 250 mL Erlenmeyer flask containing yeast extract powder (1 g/L), D-glucose (10 g/L), KH_2_PO_4_ (1 g/L), MgSO_4_.7H_2_O (5 g/L), NaCl (5 g/L), lignin (0.1 g/L) and incubated at 28 °C with 150 rpm for 14 days (Vantamuri and Kaliwal 2016). A flask containing the media without inoculum was taken as control. A reaction mixture containing 10 mM sodium acetate buffer (pH 4.5), 0.15 mM Diammonium 2, 2′-azino-bis (3-ethylbenzothiazoline-6-sulfonate) (ABTS), and 0.1 mL of extract incubated at 30 °C for 10 min. Oxidation of ABTS was monitored periodically for 14 days by measuring the increase in absorbance at 420 nm in an Evolution 202 UV vis spectrophotometer (Thermo Scientific, MA, USA). One enzyme unit is defined as the amount of enzyme that oxidizes 1 µmol of ABTS per min.

### Superoxide dismutase (SOD) assay

Superoxide dismutase activity was monitored by following the protocol of Debona et al. (2014). Mycelia from the interacting zone was weighed and crushed in phosphate buffer (pH 7.8). SOD activity was assayed on the basis of its ability to inhibit the photochemical reduction of nitro-blue tetrazolium (NBT). A reaction mixture containing 50 mM phosphate buffer (pH 7.8), 13 mM methionine, 75 mM NBT, 2 mM riboflavin, 0.1 mM EDTA, and 100 µL of extract incubated in light for 30 min was used to perform the assay. Controls which were incubated in dark were maintained for each set. Spectrophotometric readings were taken at 460 nm periodically for 14 days post inoculation.

### Quantitative real-time PCR (qRT-PCR) analysis of associated genes

A total of 14 test genes and one housekeeping gene were selected for analyzing their expression at transcript levels by qRT-PCR during the interaction of *T. coccinea* versus *T. versicolor.* Primers used in this study along with their target genes are listed in Additional file 1: Table S1. On the 8th day of interaction, total RNA of *T. coccinea* versus *T. versicolor* and their monocultures were isolated using TRI Reagent® (Sigma-Aldrich, MO, USA). The isolated RNA samples were treated with DNase I (Thermo Fisher Scientific, Waltham, MA, USA) and purified using silica column (Life Technologies, Carlsbad, CA, USA). First strand cDNA was prepared from the isolated RNA using GoScript™ Reverse Transcription kit (Promega, Madison, WI, USA). Quantitative real-time PCR was performed on the first strand cDNA using GoTaq qPCR Master Mix (Promega, Madison, WI, USA) in a total reaction volume of 20 μL containing 10 nM of each primer and 50 ng cDNA template according to the manufacturer’s protocol. Real-time PCR was performed using three biological replicates on the Quant studio 5 Real-Time PCR System (Applied Biosystems, Foster City, CA, USA) with alpha-tubulin (*atn*) gene as the reference gene. The relative gene expression of each gene was determined using the 2^−ΔΔCt^ method (Livak and Schmittgen 2001).

### Statistical analysis

The data obtained from laccase and SOD assay were analyzed in SPSS 25.0 software by one-way Analysis of Variance (ANOVA). Duncan test was performed to study the level of significance (*p* ≤ 0.05). The results of quantitative Real-time PCR were analyzed using student *t*-test in Origin Pro 6.0 and *p* ≤ 0.05 was considered as significant.

## Results

### SEM analysis during in vitro fungal-fungal interaction

**Fig. 2:**
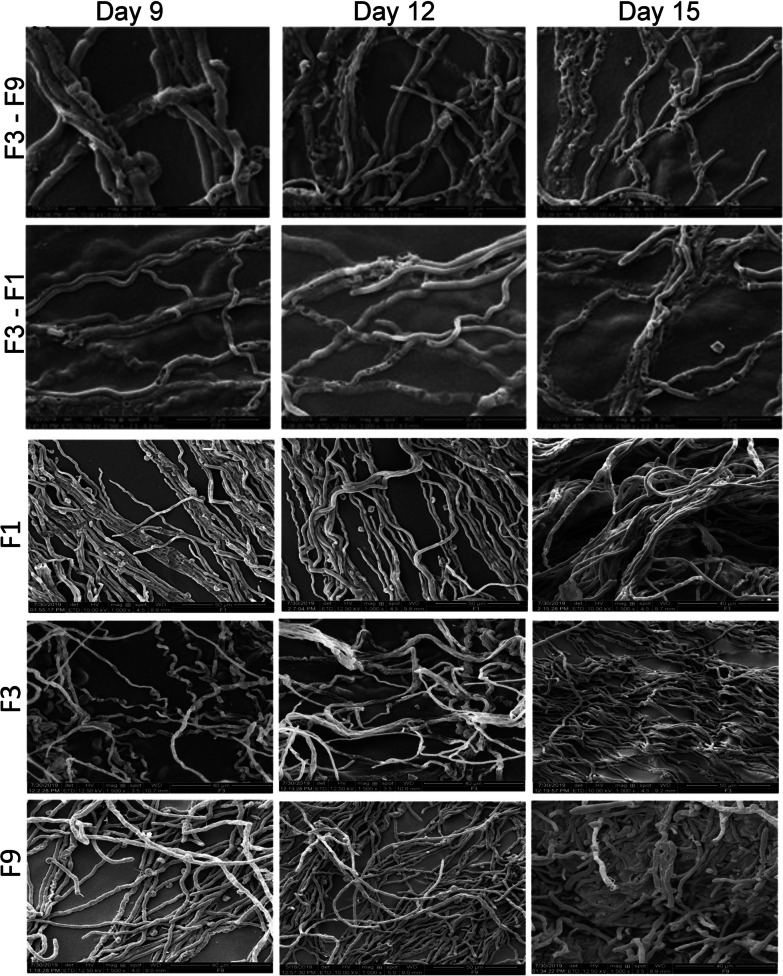
Scanning electron microscopic observations during interaction. SEM images during the interaction of *T. coccinea* with *L. lactinea*, *T. coccinea* with *T. versicolor* and the monocultures of *T. versicolor*, *T. coccinea*, *L. lactinea* respectively on 9th, 12th, and 15th days after inoculation

The SEM analysis for the interaction between *T. coccinea* and *L. lactinea* as well as *T. coccinea* and *T. versicolor* on the late stages of interaction, i.e., on the 9th, 12th and 15th days of interaction showed formation of pores on the hyphae, which was not observed for the monocultures of *T. versicolor*, *T. coccinea* and *L. lactinea* (Fig. 2). The number of pores increased with the increase in number of days of interaction, which ultimately led to the death of the hyphae in the interaction zone.

### Metabolite profiling by LC-MS

The metabolic profiling during the interaction of *T. coccinea* with *T. versicolor* along with that of the monocultures, i.e., *T. coccinea* and *T. versicolor* (Table 1) resulted in identification of 39 compounds. During the interaction 17 compounds are produced; 8 compounds were produced exclusively by *T. coccinea* monoculture and 13 were produced exclusively by *T. versicolor* monoculture. Only one compound was common between *T. coccinea* and its interaction with *T. versicolor*. Similarly, the metabolic profiling during the interaction of *T. coccinea* with *L. lactinea* along with that of the monocultures, i.e., *T. coccinea* and *L. lactinea* (Table 2) revealed the presence of 39 compounds, of which 19 compounds were produced exclusively during the interaction, 8 compounds were produced by *T. coccinea* and 10 compounds were produced by *L. lactinea*. Two compounds were common for *T. coccinea* and its interaction with *L. lactinea*. The result showed that more numbers of compounds were obtained from the barrage zone as compared to the monocultures indicating the induction of metabolite production during the interaction process. One of the significant results obtained from the LCMS and HRMS analysis is the detection of compounds like tyrosine, L-DOPA and melanin in the barrage zone (Additional files 2, 3, 4, 5: Figs. S1, S2A, S2B, S2C). Tyrosine and L-DOPA are involved in melanin synthesis pathway (Blagoeva 1984; Rzepka et al. 2016).

**Table 1:**
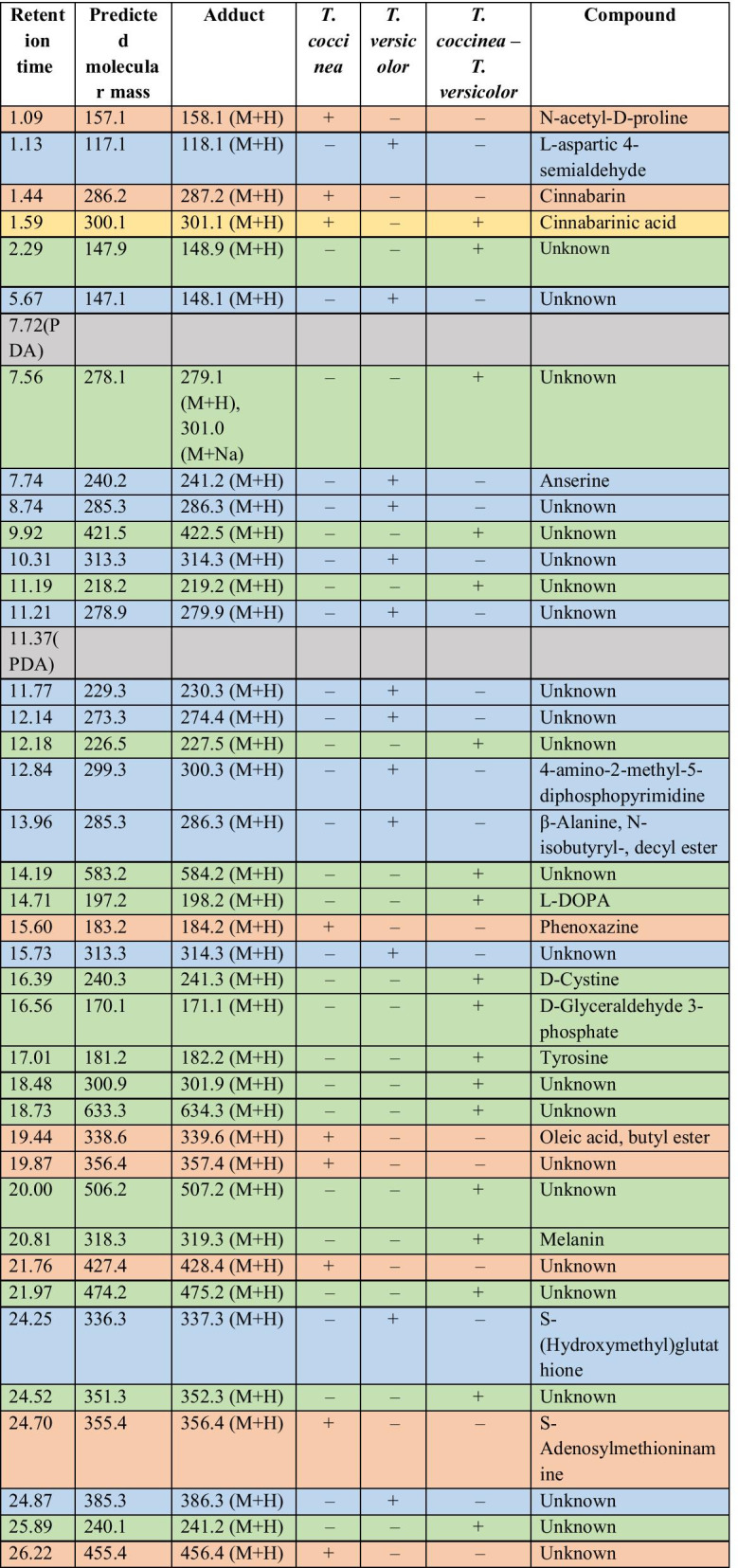
Comparative analysis of metabolite profiles during interspecific interactions between *T. coccinea*–*T. versicolor*

The ‘+’ sign indicates presence and the ‘−’ sign indicates absence of a particular compound. The orange-coloured cells are for the compounds produced exclusively by *T. coccinea*; the blue-coloured cells are for the compounds produced exclusively by *T. versicolor*; the yellow-coloured cells are for the compounds present in either or both the monocultures and in the interaction zone, the green-coloured cells are for the compounds produced exclusively in the interaction zone; the grey-coloured cells are for the components of PDA that has been omitted for analysis

**Table 2:**
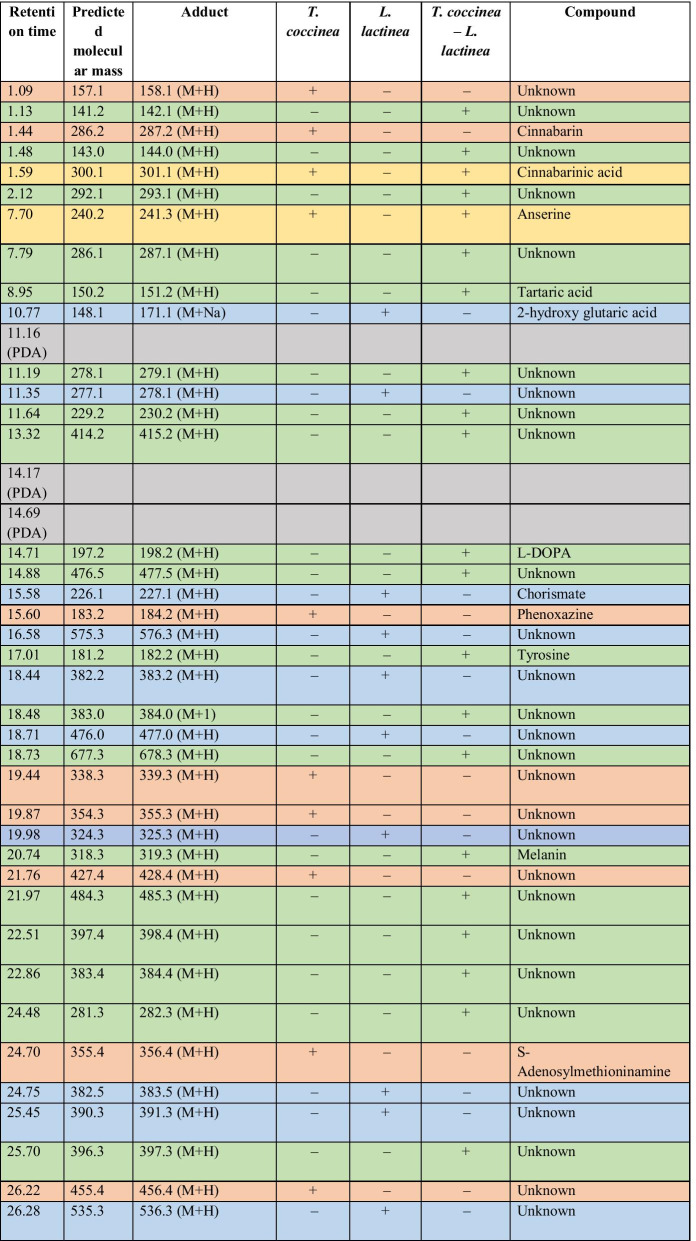
Comparative analysis of metabolite profiles during interspecific interactions between *T. coccinea*–*L. lactinea*

The ‘+’ sign indicates presence and the ‘−’ sign indicates absence of a particular compound. The orange-coloured cells are for the compounds produced exclusively by *T. coccinea*; the blue-coloured cells are for the compounds produced exclusively by *L. lactinea*; the yellow-coloured cells are for the compounds present in either or both the monocultures and in the interaction zone, the green-coloured cells are for the compounds produced exclusively in the interaction zone; the grey-coloured cells are for the components of PDA that has been omitted for analysis

### Extraction of extracellular melanin pigment

**Fig. 3:**
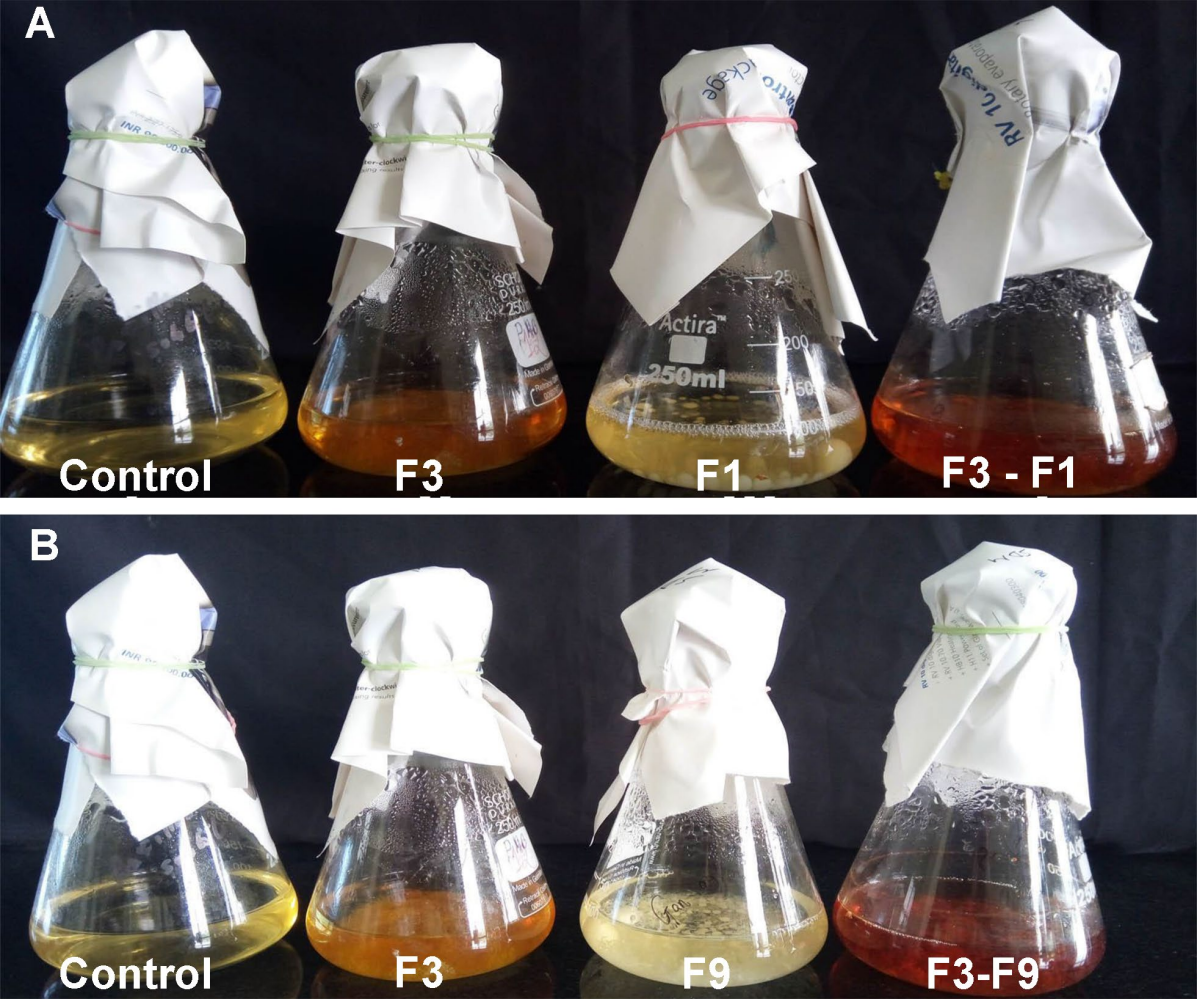
Culture broth showing melanin production. **A**, **B** Dark brown formation in the flask containing the interacting mycelia for *T. coccinea* versus *T. versicolor* and *T. coccinea* versus *L. lactinea* respectively

The SB broth where the two different fungi were dual cultured (i.e., *T. coccinea* and *T. versicolor* (Fig. 3A); *T. coccinea* and *L. lactinea* (Fig. 3B) showed dark brown coloration. On the other hand, no dark brown coloration was observed in the control and the monocultures of *T. coccinea*, *T. versicolor,* and *L. lactinea*. As the color of the culture supernatant in control remained unchanged, it could be assumed that the dark coloration in the interaction culture was due to the compounds secreted by the fungi and not due to the result of the oxidation of the media components. An amount of 37 mg/L and 31 mg/L of extracellular pigment were obtained as a product from the culture broth, where *T. coccinea*—*T. versicolor* and *T. coccinea—L. lactinea* were allowed to interact respectively.

### Biochemical analysis of the melanin pigment

The solubility test of the pigment showed that it was insoluble in water and organic solvents like ethanol, hexane, and acetone but soluble in 1 M KOH and 1 M NaOH. Also, the brown coloration of the pigment faded away when it was treated with an oxidizing agent like hydrogen peroxide. Treatment of melanin with hydrochloric acid resulted in precipitate formation (Table 3).

**Table 3:** Biochemical characterization of melanin

Sl. no	Assay	Result
1	Solubility in water	Insoluble
2	Solubility in ethanol	Insoluble
3	Solubility in hexane	Insoluble
4	Solubility in acetone	Insoluble
5	Solubility in 1 M KOH	Soluble
6	Solubility in 1 M NaOH	Soluble
7	Reaction with hydrogen peroxide	Decolorization of the pigment
8	Reaction with hydrochloric acid	Precipitated readily

### HPLC analysis

The High-Performance Liquid Chromatography analysis showed that the chromatograms of synthetic melanin and brown pigment extracted from the barrage zones are composed of a single major signal with > 90% purity. The retention time is ~ 5.06 min for both synthetic- and fungal- melanin (Additional file 6: Fig. S3).

### Characterization of the melanin pigment from FTIR spectrum

**Fig. 4:**
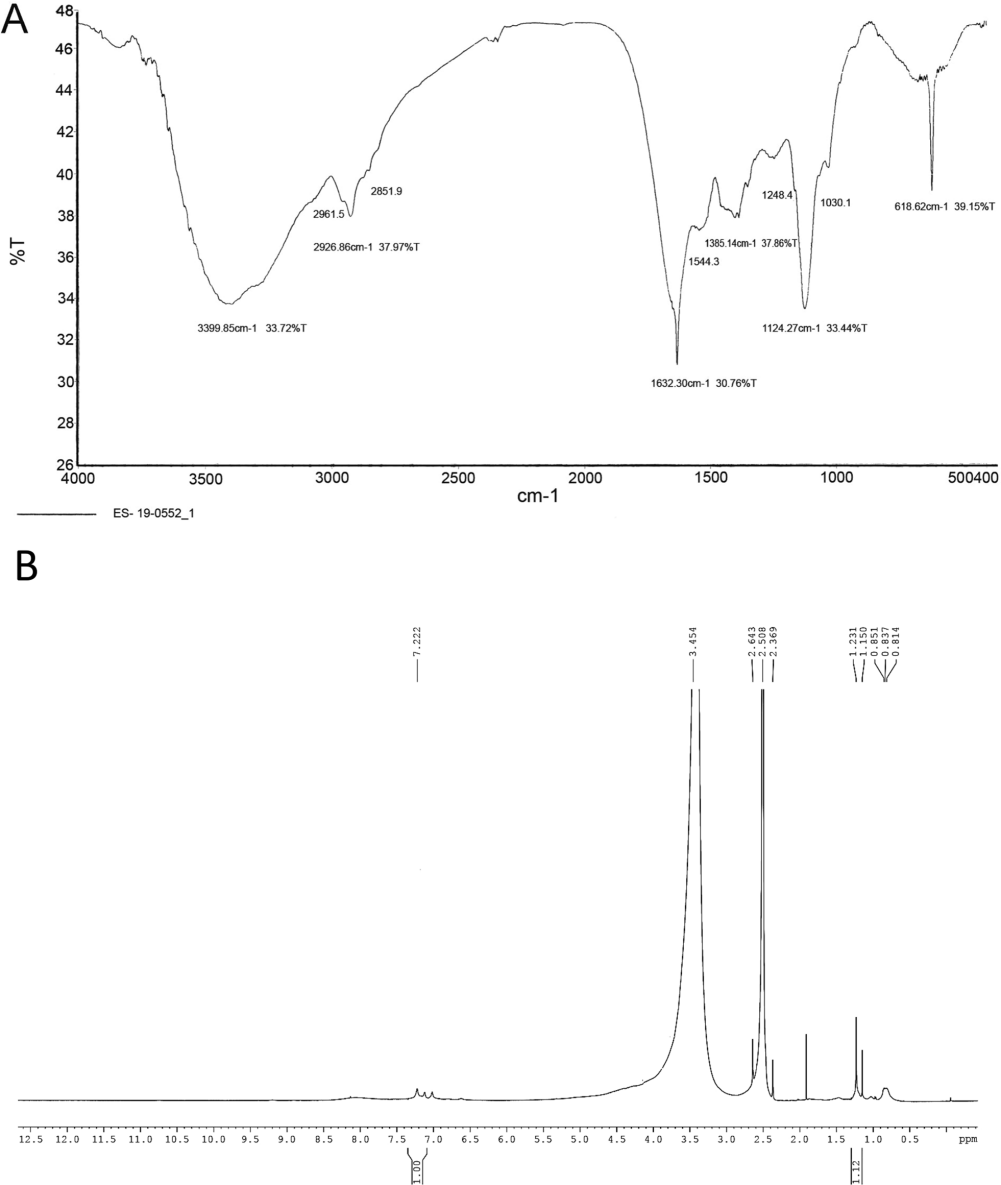
Spectroscopic characterization of melanin pigment purified from *T. coccinea*—*T. versicolor culture.* **A** FTIR spectrum. **B** NMR spectrum

In FTIR spectrum, peaks were obtained at 3399 cm^−1^, 2,926 cm^−1^, 2,851 cm^−1^, 1,586 cm^−1^, 1385 cm^−1^, 1030 cm^−1^ and 618 cm^−1^. Absorption at 3399 cm^−1^ attributes to the polymeric OH groups. The stretching vibrations for aliphatic CH bonding appear at 2926 cm^−1^ and 2851 cm^−1^. At 1586 cm^−1^, the symmetric carboxylate stretching vibrations (COO) are detectable. The indole ring vibration/CNC stretching was observed at 1385 cm^−1^. CH in-plane/CH out-of plane deformation are attributed at 1030 cm^−1^. 618 cm^−1^ indicates to the out-of-plane bending of the aromatic carbon-hydrogen bond (Fig. 4A). The presence of these functional groups indicates the presence of melanin.

### Characterization of the melanin pigment from NMR spectrum

In NMR spectrum, peaks were obtained at 0.814 ppm, 0.837 ppm, 0.851, 1.150 ppm, 1.231 ppm, 2.369 ppm, 2.508 ppm, 2.643 ppm, 3.454 ppm and 7.222 ppm. 0.814–1.150 ppm can be attributed to the CH_3_ groups of alkyl fragments. Peak at 1.231 ppm can be attributed to the long chain methylenes. Peak at 2.369 ppm is associated with the CH at aliphatic region. The signal at 2.508 ppm is associated with DMSO, which comes from the solvent deuterated DMSO or sulfonate groups bound to the pyrrole nitrogen which is relative to the occurrence of N-sulfonation. Peak at 7.222 ppm is related to the pyrrole –CH group of a carboxyl substituted indole. A peak ranging at 3.454 ppm is also observed tentatively near the signal from residual water in the DMSO. Peak at 2.643 ppm indicates CH_2_CO group (Fig. 4B). The presence of these functional groups infers the compound to be melanin.

### Assessment of laccase activity during in vitro fungal-fungal interaction

**Fig. 5:**
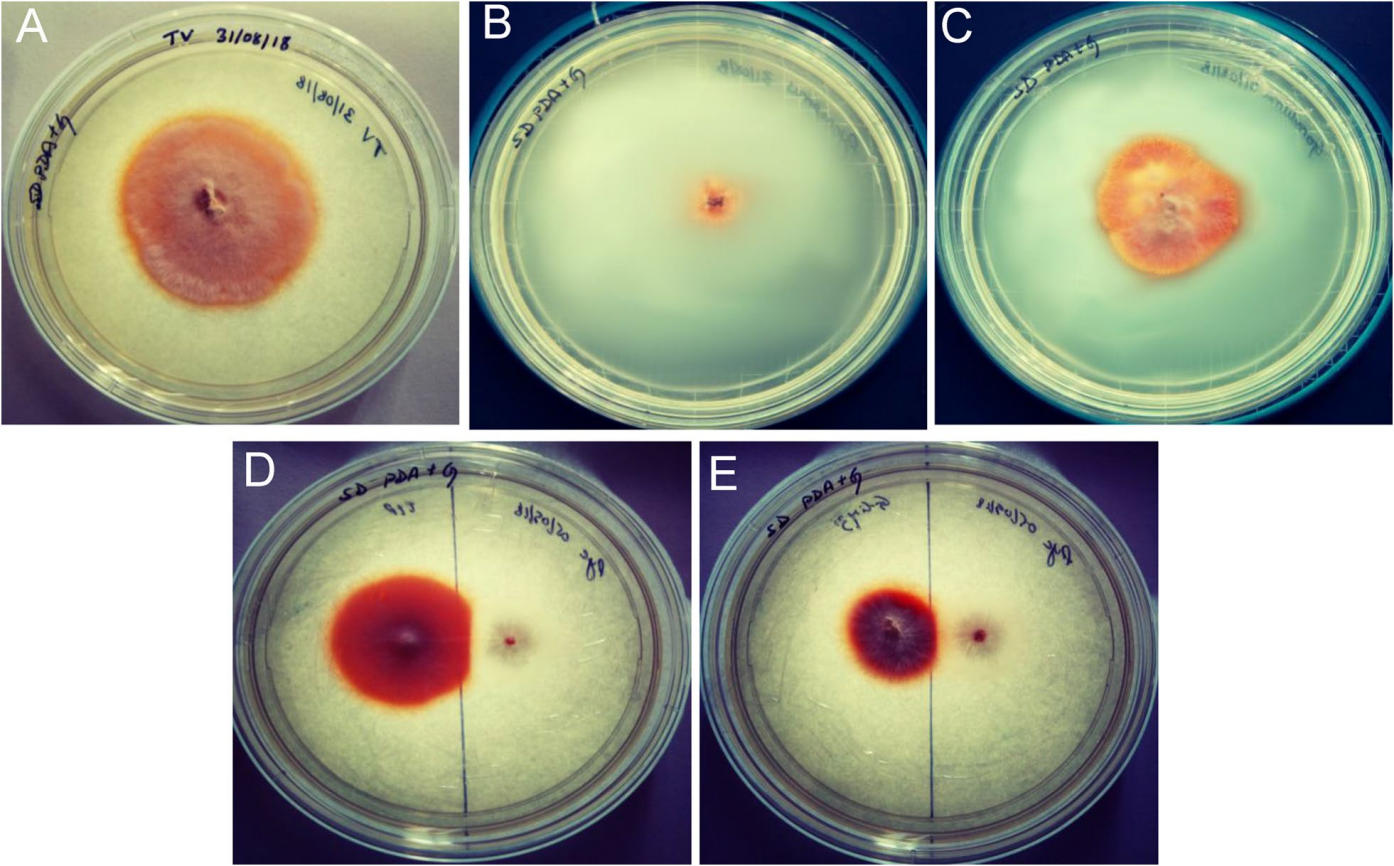
Qualitative laccase activity for plate cultures. **A** *T. versicolor* (F1). **B** *T. coccinea* (F3). **C** *L. lactinea* (F9). **D** *T. coccinea* versus *T. versicolor.* **E** *T. coccinea* versus *L. lactinea*

**Fig. 6:**
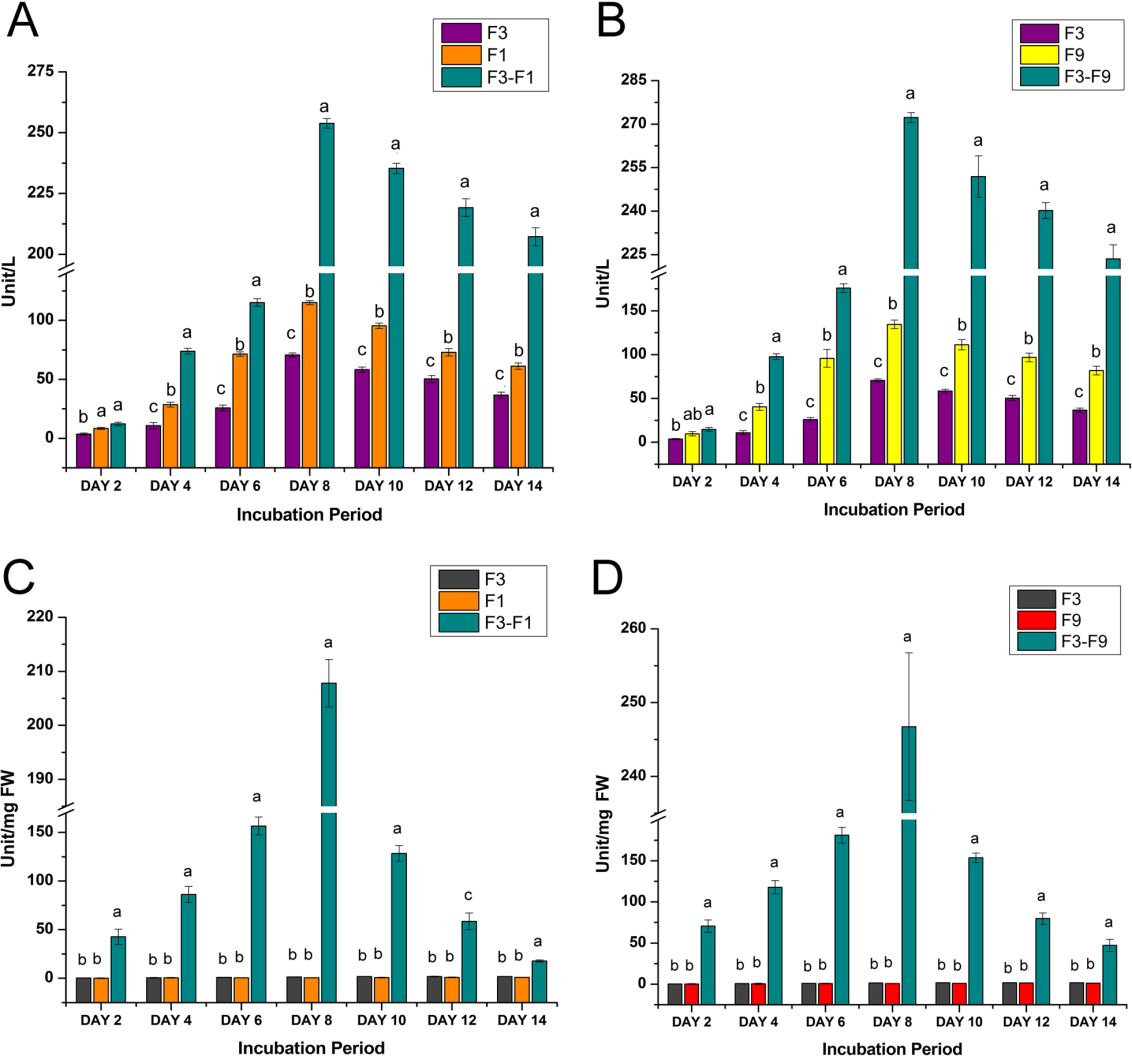
Quantitative analysis for enzyme production. **A** Laccase production by *T. coccinea* (F3), *T. versicolor* (F1) and their interaction (F3–F1). **B** Laccase production by *T. coccinea* (F3), *L. lactinea* (F9) and their interaction (F3–F9). **C** SOD production by *T. coccinea* (F3), *T. versicolor* (F1) and their interaction (F3–F1). **D** SOD production by *T. coccinea* (F3), *L. lactinea* (F9) and their interaction (F3–F9) (level of significance with *p* ≤ 0.05)

Qualitative analysis of laccase activity through plate assay indicated *T. coccinea*, *T. versicolor* and *L. lactinea* to be laccase positive (Fig. 5 and Additional file 7). Quantification of laccase activity revealed that the interactions increased laccase activities as compared to the enzyme activity in pure cultures. The laccase activity increased from 2nd day post inoculation up to the 8th day, and then slightly decreased on the succeeding days until the 14th day post inoculation for both the interactions (Fig. 6A, B). The laccase activity in case of the pure cultures also increased till the 8th days post inoculation but was less (significantly low), compared to the interaction ones. The laccase activity for *T. coccinea* with *T. versicolor* (F3–F1) and *T. coccinea* with *L. lactinea* (F3–F9) in the interaction zone was found to be 253.86 U/L and 272.25 U/L respectively. This was more as compared to the laccase activity of the monocultures of *T. coccinea*, *T. versicolor* and *L. lactinea* was found to be 70.64 U/L, 115.03 U/L and 134.42 U/L (*p* ≤ 0.05).

### Assessment of superoxide dismutase (SOD) activity during in vitro fungal-fungal interaction

The superoxide dismutase assay showed that during the initial days of interaction (4th, 6th and 8th days) the superoxide dismutase activity was higher for the interacting fungi. But their activity gradually decreased from the 10th day post inoculation. Superoxide dismutase activity was very negligible as observed for the monocultures i.e., 1.5 U/mgFW in *T. coccinea*, 0.57 U/mgFW in *T. versicolor* and 0.79 U/mgFW in *L. lactinea* (Additional file 7﻿). The SOD activity for *T. coccinea* with *T. versicolor* (F3–F1) and *T. coccinea* with *L. lactinea* (F3–F9) in the interaction zone was found to be highest, i.e., 207.81 U/mgFW and 246.74 U/mgFW respectively at *p* ≤ 0.05 (Fig. 6C, D).

### Gene expression profile during interaction of *T. coccinea* and *T. versicolor*

**Fig. 7:**
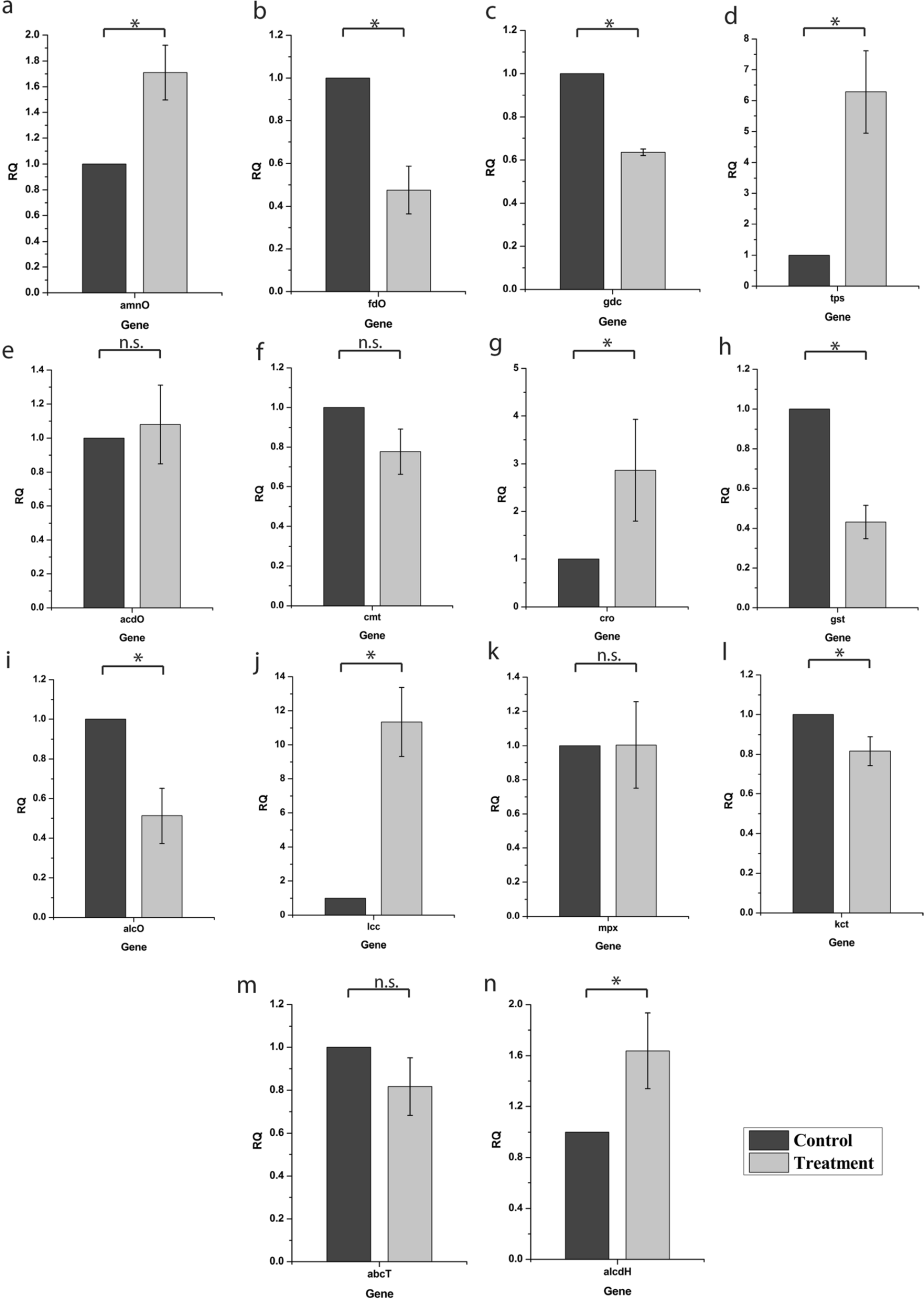
qRT-PCR analysis of *T. versicolor* (control) and *T. coccinea* versus *T. versicolor* (treatment). **A** Amine oxidase. **B** FAD—linked oxidoreductase. **C** Glutamate decarboxylase. **D** Terpenoid synthase. **E** Alcohol dehydrogenase. **F** CDF metal transporter. **G** Copper radical oxidase. **H** Glutathione-S-transferase. **I** Alcohol oxidase. **J** Laccase. **K** Manganese peroxidase. **L** 3-ketoacyl-CoA thiolase. **M** ABC-transporter. **N** Alcohol dehydrogenase. Significance was calculated using student’s t test with *p* ≤ 0.05

Quantitative Real time PCR (qRT-PCR) analysis of the 14 genes during interaction of *T. coccinea* and *T. versicolor* was performed and compared to the expression profile of *T. versicolor* control culture to understand how the interaction of the two fungal isolates affect the expression of these genes. The results of qRT-PCR analysis are shown in Fig. 7 and Additional file 8. Among the genes that showed upregulation during interaction, the *lcc1* gene that encodes laccase showed 11.1-fold increased expression during interaction followed by *tps* (6.2-fold), *cro* (2.8 folds), *amnO* (1.7-fold) and *alcDH* (1.6-fold). Among the genes tested, *fdo* (gene encoding FAD-linked oxidoreductase), *gdc* (gene encoding glutamate decarboxylase), *gst* (gene encoding glutathione S transferase), *alcO* (gene encoding alcohol oxidase) and *kct* (gene encoding 3-ketoacyl-CoA thiolase) showed significantly decrease in expression during interaction. The rest of the genes viz., *abcT* (gene encoding an ABC transporter), *acdo* (gene encoding aromatic compound dioxygenase), *cmt* (gene encoding CDF metal transporter), and *mpx* (gene encoding manganese peroxidase) did not reveal any significant difference in their expression during interaction of *T. coccinea* and *T. versicolor*.

## Discussion

Fungal melanins are secondary metabolite made up of complex heterogeneous polymers of phenolic and/or indole monomers. They are reported to play a myriad of biological roles such as in morphogenesis, virulence, energy transduction including acting as scavengers of stresses, and in turn protect cell viability (Toledo et al. 2017). Previously, brown pigmentation was reported in the barrage zone of fungal interactions due to deposition of certain quinone compounds assumed to be melanin or melanin like compounds and is associated with morphological changes at interacting hyphal fronts (Peiris et al. 2008). However, interesting changes were observed in the present study during deadlock interaction, where the interacting hyphae showed altered hyphal morphology and pigment production. To explore these interactions fully, the changes in hyphal morphology, metabolite production and gene expression were studied. The occurrence of pigmentation in the barrage zone could be correlated to the deposition of melanin pigments which got induced during stress condition due to the physical contact between the competing hyphae. HPLC and LC-MS analysis suggested the presence of melanin as a major compound in the zones of interactions. Biophysical analysis using FTIR and NMR further confirmed that the brown pigment is melanin. The amount of melanin produced during fungal-fungal interaction in the present study was higher (31–37 mg/L) when compared to the melanin produced by monocultures of different species as compared to the previous study on *Schizophyllum commune*, which produced only 0.25 mg/L of melanin from the culture broth (Arun et al. 2015).

Morphological changes were also observed in the zone of interaction which is often related to altered metabolism conferred by various metabolic and oxidative enzymes, increase or decrease of which could lead to a series of other changes. Scanning electron microscopic analysis of fungal hyphae from interaction zone revealed formation of pores on the hyphae of the interacting isolates leading to distortion of the hyphae and culminated in death of hyphae which can be attributed as a characteristic feature of deadlock interaction. In many studies, various authors have mentioned that in case of deadlock interaction, both the interacting fungi compete strongly so that neither of the fungi can acquire the territory of the other (Boddy 2000). One or both of the interacting fungi secrete some important metabolites which results in the death of the interacting hyphae in the interaction zone (Peiris et al. 2008). Therefore, the changes in metabolite profile during the interaction of *T. coccinea* with *T. versicolor* and *T. coccinea* with *L. lactinea* was analyzed. The metabolic profile revealed that during the interaction process many compounds were synthesized or induced which were otherwise not produced by the mono cultures. The presence of compounds like tyrosine and L-DOPA could be co-related to the production of melanin—an important bioactive molecule (Slominski et al. 2012; Luo et al. 2017; Almeida-Paes et al. 2018).

In general, there is also a possibility of competition between fungal species for nutritional and habitat resources resulting in the induction of stress related compounds as well as laccase activity (Gregorio et al. 2006). The enzyme is also reported to play a crucial role in detoxification. The increase in laccase activity in the interaction zone may be due to the defense reaction resulting from mycelial confrontations (Baldrian 2004). Eyre et al. (2010) reported that enzymes involved in reactive oxygen species (ROS) generation, like the NADPH oxidases, laccase and peroxidases are occasionally upregulated during interaction. Accumulation of ROS in barrage zones causes oxidative damage to competitor mycelia, particularly in the deadlock ones. Laccase not only could function as antioxidants to remove ROS for fungal survival, but also showed a strong ability of xenobiotic detoxification as reported earlier (Zhong et al. 2019). In the present study, the increase in superoxide dismutase in the early days of post inoculation indicates the increase production of ROS. Reports suggested that ROS production increases in the interaction zone due to oxidative stress during the interaction of *Dichomitus squalens* and *P. ostreatus*. This increase in ROS production results in the activation of first line of defense by enhancing the activity of superoxide dismutase which helps in scavenging the ROS compounds (Tamayo et al. 2016).

Morphological changes are associated with changes in gene expression compared to non-interacting mycelia during interactions. For example, during antagonistic interaction, downregulation of chitin synthase in *S. gausapatum*; there is decrease in growth with its possible replacement by *T. versicolor* (Eyre et al. 2010). Quantitative real-time PCR suggested that several stress related genes from *T. versicolor* were differentially expressed during the interaction process. We could not study the differential gene expression of *T. coccinea* during interaction due to the unavailability of completely annotated genome sequence of *T. coccinea*. The *lcc1* gene of *T. versicolor*, which encodes the laccase enzyme showed significant upregulation (*p* ≤ 0.05) compared to the monoculture. Laccases have been reported earlier to be involved in melanin production (Boddy and Hiscox 2016). Although many studies have revealed that interspecific fungal interactions contribute to the increase in laccase activity, (Baldrian 2004; Chi et al. 2007; Gregorio et al. 2006; Hiscox et al. 2010; Kuhar et al. 2015; Wei et al. 2010) the mechanism of laccase production caused by mycelial interactions still remains elusive. Here, the production of melanin in the interaction culture (as revealed by LC-MS, FTIR and NMR analysis) could be correlated to the high laccase activity, which was due to the upregulation of *lcc1* gene. Other oxidative stress related genes, such as *tps* (gene encoding terpenoid synthase), *cro* (gene encoding copper radical oxidase), *amnO* (gene encoding amine oxidase) and *alcDH* (gene encoding alcohol dehydrogenase) were also induced during interaction, which was at par the earlier report (Zhong et al. 2019). Differential expression of these stress related genes indicated the occurrence of stress signals during the interspecific mycelial interaction of the two macrofungal species (Silar 2005).

## Conclusion

The present study revealed that the changes in the hyphal pattern during the fungal interaction among the three white rot fungi are mostly related to the metabolites produced during interaction. An overproduction of the extracellular enzyme laccase during the fungal interaction was also marked to have a role in the detoxification process. This may be due to the production of some toxic compounds or may be due to the production of ROS during interaction by one or both of the interacting fungi. As a result of this, the production of laccase in the interaction zone increases by either or both the fungi. The findings of this study are further supported by the evidence of increase superoxide dismutase activity that acts as a scavenging radical. The present study analysed differential genes expression during interspecific fungal interactions, revealing that defense-related responses and a myriad of signaling pathways might be associated with the upregulation of oxidative stress-resistant genes, along with the production of an industrially important bioactive compound (melanin), and thereby competing for both nutrient and territory.

## Supplementary Information

**Additional file 1: Table S1.** List of oligonucleotide primers used in the present study.

**Additional file 2: Fig. S1.** LC-MS chromatogram of the extracts. **A**. *Trametes coccinea*. **B**. *Trametes versicolor*. **C**. *Leiotrametes lactinea*., **D**. *T. coccinea* *vs*. *T. versicolor*. **E**. *T*. *coccinea vs. L. lactinea.*

**Additional file 3: Fig. S2A.** HRMS spectra of Tyrosine.

**Additional file 4: Fig. S2B.** HRMS spectra of L-DOPA.

**Additional file 5: Fig. S2C.** HRMS spectra of Melanin.

**Additional file 6: Fig. S3.** TLC analysis of melanin. A. Melanin extracted from fungal dual culture. B. Standard Melanin (Sigma, USA).

**Additional file 7:** Lacccase and Superoxide dismutase (SOD) activity of *T. coccinea—T. versicolor* and *T. coccinea—L. lactinea *dual cultures as well as their monocultures.

**Additional file 8:** Result of qRT-PCR analysis.

## Data Availability

The datasets used and/or analysed during the current study are available from the corresponding author on reasonable request.
