# Supplementary material for: Melanin production and laccase mediated oxidative stress alleviation during fungal-fungal interaction among basidiomycete fungi
Source: IMA Fungus. 2021 Nov 9;12:33. doi: 10.1186/s43008-021-00082-y (PMC8576908; doi:10.1186/s43008-021-00082-y)
Supplement: Supplementary file 1 — Additional file 1: Table S1. List of oligonucleotide primers used in the present study. [file 43008_2021_82_MOESM1_ESM.docx]

**Table S1-** **List of oligonucleotide primers used in the quantitative real time PCR analysis**

| **Gene/Locus** | **Product** | **Primer** | **Sequence (5'->3')** | **Length (bp)** |
| --- | --- | --- | --- | --- |
| TRAVEDRAFT_29635 | Alcohol dehydrogenase | AlcDH F | GGGCAAACGGTCCTCATCTT | 20 |
|  |  | AlcDH R | GTAGTCCGCCCCAAGAGAAC | 20 |
| TRAVEDRAFT_144610 | Alcohol Oxidase | AlcO F | CGTGGTGCTTGGTCCTACAT | 20 |
|  |  | AlcO R | GCAGGTCTTTGACCACCGTA | 20 |
| TRAVEDRAFT_128490 | Amine Oxidase | AmnO F | TTACGGAGGACCTTTTCGGC | 20 |
|  |  | AmnO R | AAGAAGTTGACGGGGTGCAA | 20 |
| TRAVEDRAFT_73942 | Glutathione-S-transferase | GST F | ATCGAGACGGCCAACTTTGA | 20 |
|  |  | GST R | TCATCGCCAGCAAGGAACTT | 20 |
| lcc1 | Laccase | LCC F | GGTGGGATTAACTCCGCCAT | 20 |
|  |  | LCC R | AAGACCATGTTCAGCGGTGT | 20 |
| VP3at | Manganese Peroxidase | MPX F | TGGCTTTCAAAACTCTCGCC | 20 |
|  |  | MPX R | CGAACAGGTTCTGCTGGATG | 20 |
| TRAVEDRAFT_158338 | 3-Ketoacyl-CoA thiolase | KCT F | ATGGCACGACGAAGGAGAAC | 20 |
|  |  | KCT R | AGTCGTGACATGCTTTGCCA | 20 |
| TRAVEDRAFT_31517 | ABC-transporter | ABCT F | TTGGGCTTCATTTTCGCCAC | 20 |
|  |  | ABCT R | CGAGTGGACCCAACGAAGAA | 20 |
| TRAVEDRAFT_45420 | Copper Radical Oxidase | CRO F | GATGAGTGCTGGTGTGGTGA | 20 |
|  |  | CRO R | ATAGAGCTGCAACCGTGCTT | 20 |
| TRAVEDRAFT_75578 | Terpenoid Synthase | TPS F | GCTCCACATAAACCGCCTCT | 20 |
|  |  | TPS R | CAGCGACGCTACATGGATCA | 20 |
| TRAVEDRAFT_139368 | CDF Metal transporter | CMT F | TTCTCATGGGCTCGGTCAAC | 20 |
|  |  | CMT R | AACTTGACACCTAGAGCCGC | 20 |
| TRAVEDRAFT_73497 | Glutamate decarboxylase | GDC F | AGCGGGATGTTCACAGTGTT | 20 |
|  |  | GDC R | TTTGAACGCCACTACGGGAA | 20 |
| TRAVEDRAFT_26075 | FAD – linked oxidoreductase | FDO F | AGGGGTGCGGCTTATATTCG | 20 |
|  |  | FDO R | GGCCTCCTTGTACCAAACCA | 20 |
| TRAVEDRAFT_28066 | Aromatic compound dioxygenase | ACDO F | GGCAAAATTCTCGCGAACCC | 20 |
|  |  | ACDO R | TATCCAGGGGCCTTGACTGT | 20 |
| TRAVEDRAFT_69843 | α-Tubulin | ATN F | AGAACAGGCTCAACGTCTCG | 20 |
|  |  | ATN R | ACGGGGAAATGAATACGGGG | 20 |
