# Supplementary figures and images for: Melanin production and laccase mediated oxidative stress alleviation during fungal-fungal interaction among basidiomycete fungi
Source: IMA Fungus. 2021 Nov 9;12:33. doi: 10.1186/s43008-021-00082-y (PMC8576908; doi:10.1186/s43008-021-00082-y)

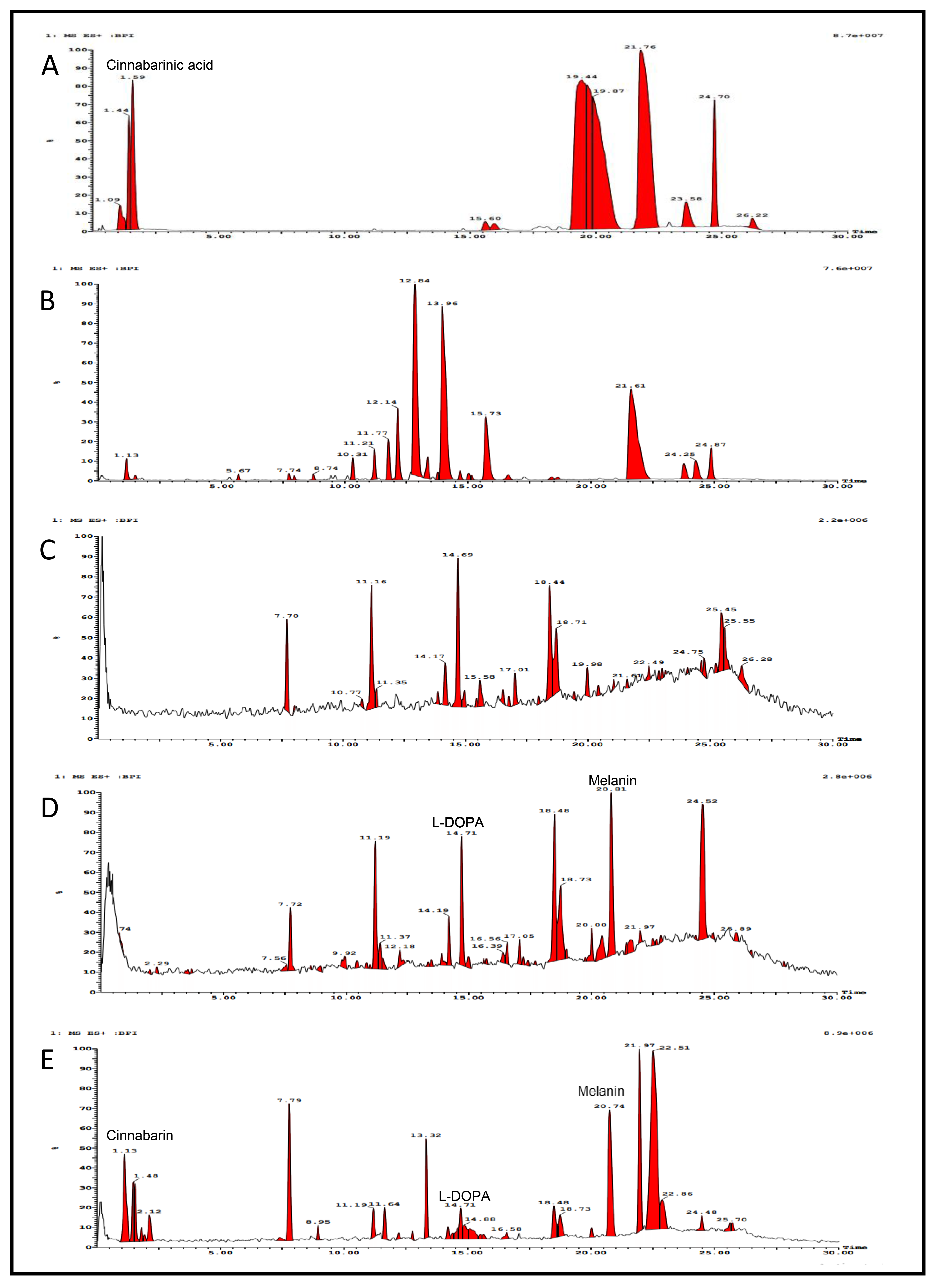

Supplement: Supplementary file 2 — Additional file 2: Fig. S1. LC-MS chromatogram of the extracts. A. Trametes coccinea. B. Trametes versicolor. C. Leiotrametes lactinea., D. T. coccinea vs. T. versicolor. E. T. coccinea vs. L. lactinea. [file 43008_2021_82_MOESM2_ESM.tif]

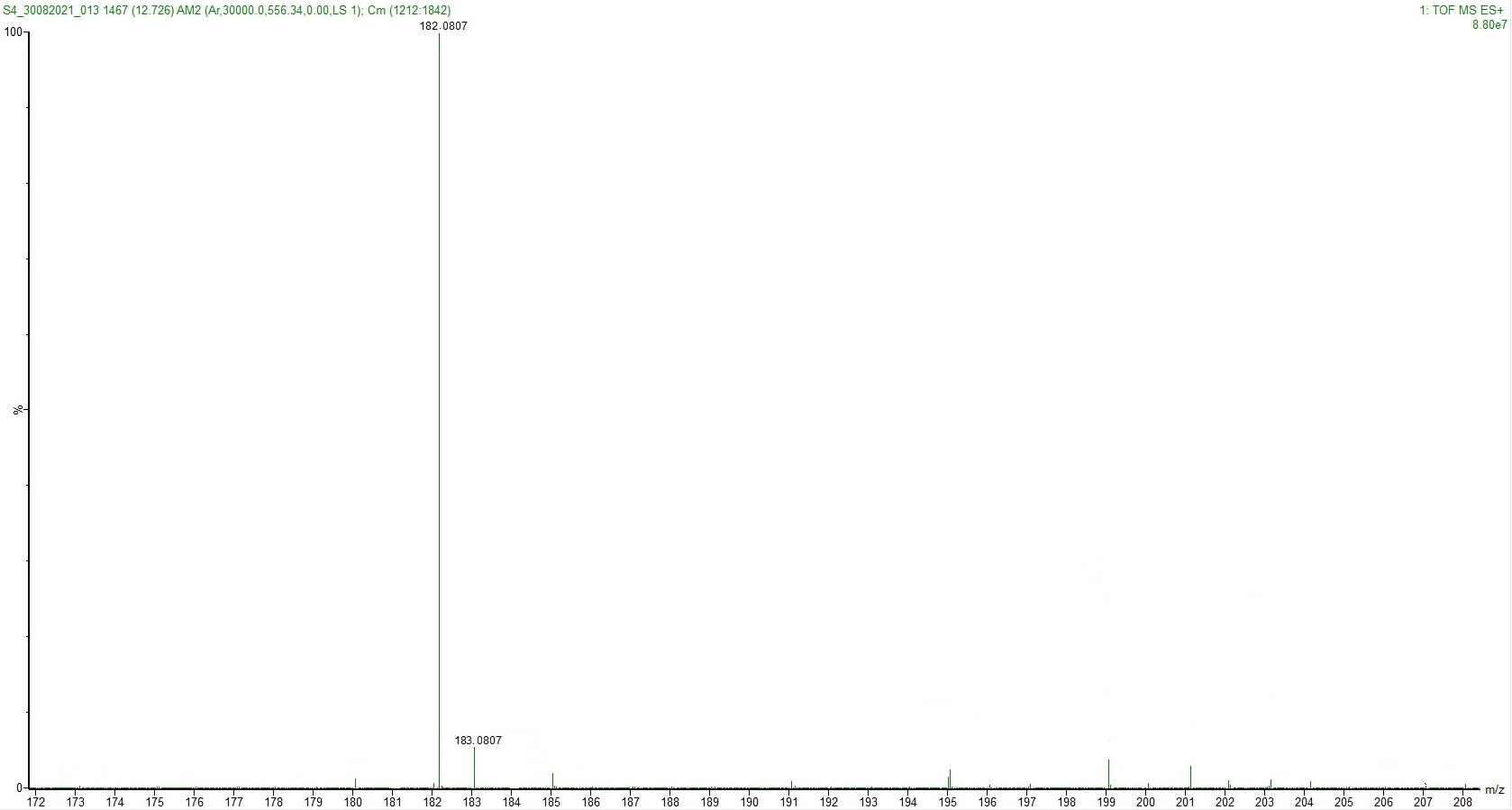

Supplement: Supplementary file 3 — Additional file 3: Fig. S2A. HRMS spectra of Tyrosine. [file 43008_2021_82_MOESM3_ESM.tif]

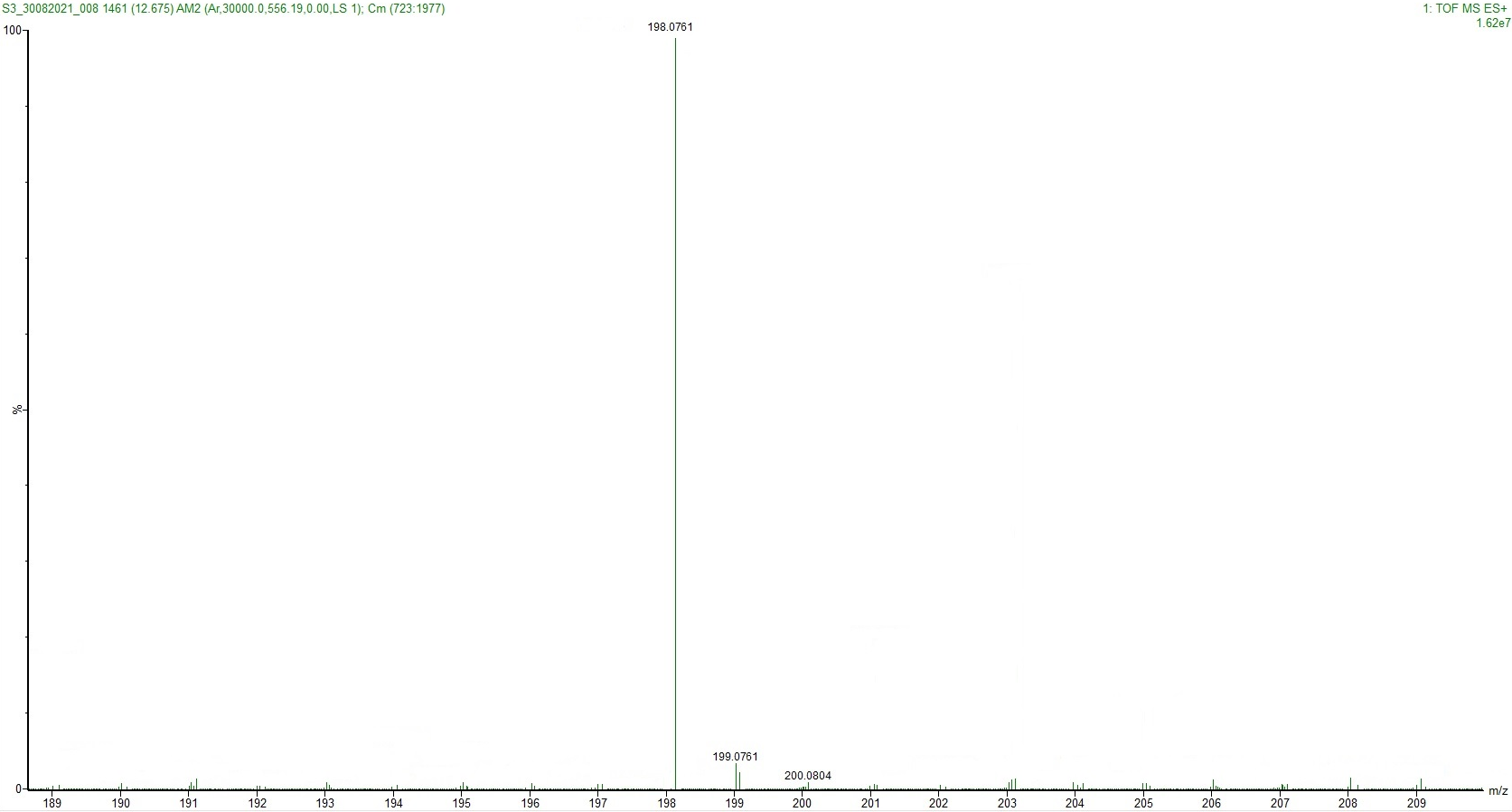

Supplement: Supplementary file 4 — Additional file 4: Fig. S2B. HRMS spectra of L-DOPA. [file 43008_2021_82_MOESM4_ESM.tif]

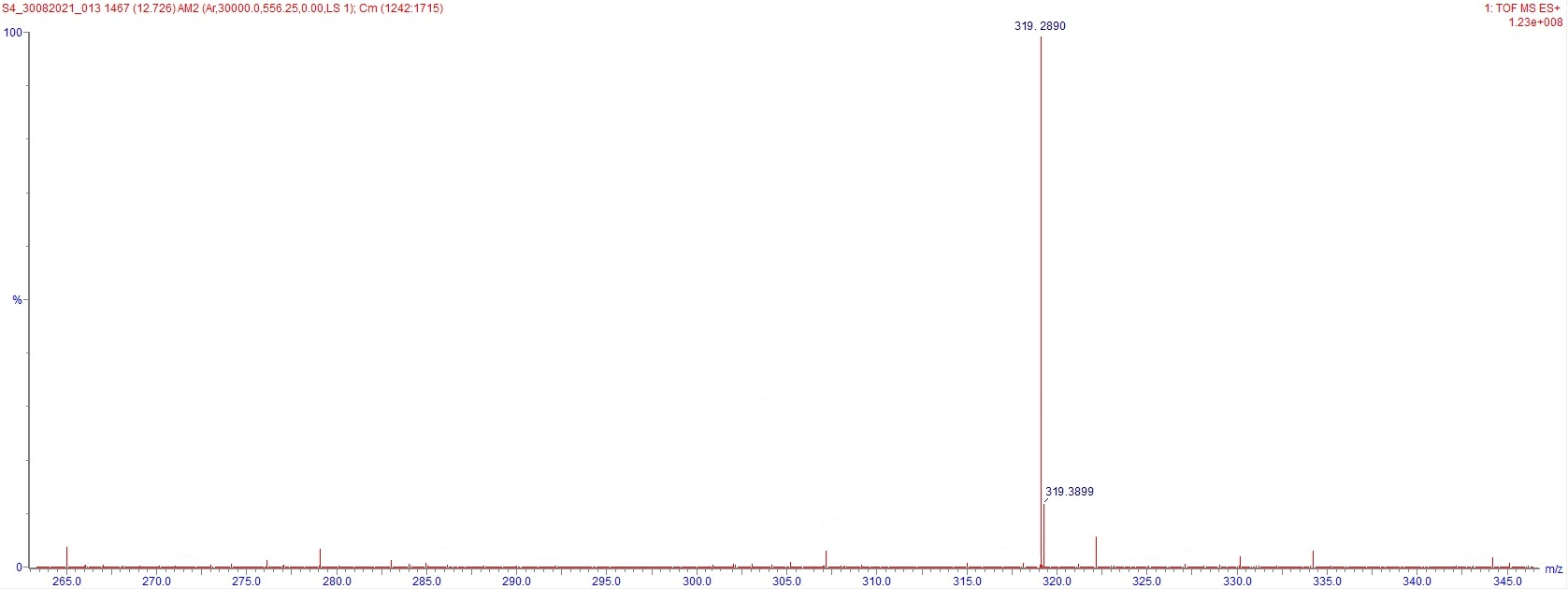

Supplement: Supplementary file 5 — Additional file 5: Fig. S2C. HRMS spectra of Melanin. [file 43008_2021_82_MOESM5_ESM.tif]

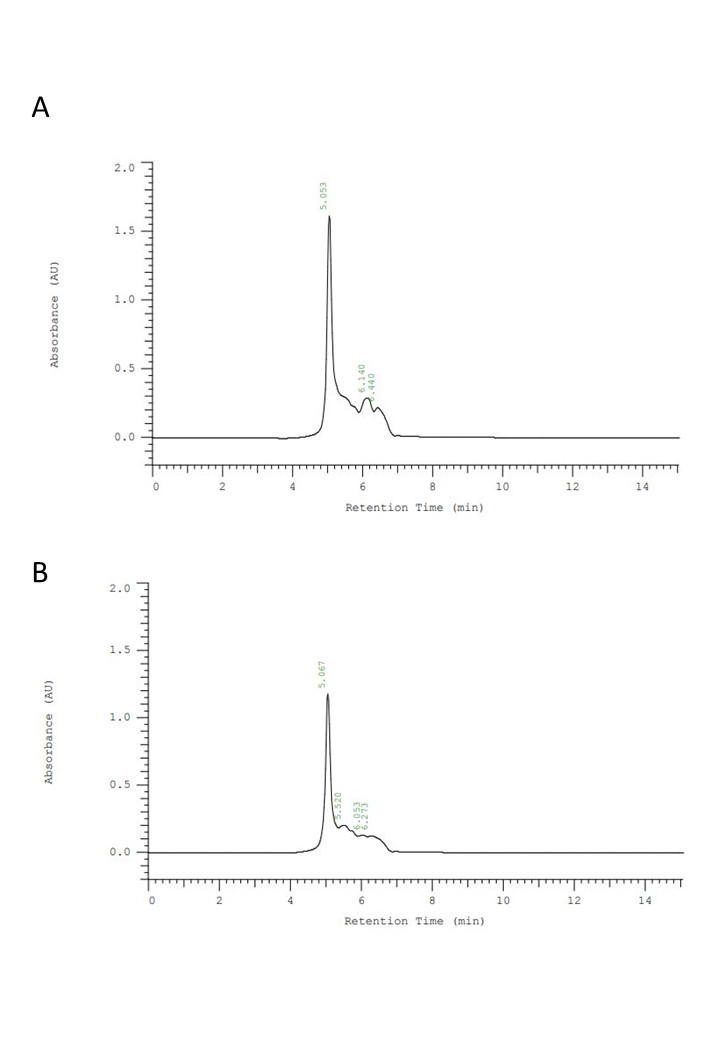

Supplement: Supplementary file 6 — Additional file 6: Fig. S3. TLC analysis of melanin. A. Melanin extracted from fungal dual culture. B. Standard Melanin (Sigma, USA). [file 43008_2021_82_MOESM6_ESM.jpg]
